# Supplementary material for: Association between albumin-corrected anion gap and in-hospital mortality of intensive care patients with trauma: A retrospective study based on MIMIC-Ⅲ and Ⅳ databases
Source: PLoS One. 2024 Mar 7;19(3):e0300012. doi: 10.1371/journal.pone.0300012 (PMC10919588; doi:10.1371/journal.pone.0300012)
Supplement: S1 Data — (ZIP) [file pone.0300012.s001.zip › Raw data/ICD codes related to trauma in the mimic databases.docx]

**All trauma-related ICD codes, including ICD-9/10, present in the MIMIC-III/IV databases**

ICD-9 codes:

80000, 80001, 80002, 80003, 80006, 80009, 80010, 80011, 80012, 80013, 80015, 80016, 80020, 80021, 80022, 80023, 80024, 80025, 80026, 80030, 80031, 80032, 80034, 80035, 80036, 80046, 80050, 80051, 80052, 80056, 80060, 80061, 80065, 80070, 80071, 80072, 80075, 80076, 80080, 80081, 80085, 80091, 80100, 80101, 80102, 80105, 80106, 80109, 80110, 80111, 80112, 80113, 80114, 80115, 80116, 80119, 80120, 80121, 80122, 80123, 80124, 80125, 80126, 80129, 80130, 80131, 80132, 80134, 80135, 80136, 80140, 80141, 80142, 80144, 80145, 80146, 80150, 80151, 80152, 80156, 80160, 80161, 80162, 80164, 80165, 80170, 80171, 80172, 80174, 80175, 80176, 80180, 80182, 80191, 80196, 8020, 8021, 80220, 80221, 80222, 80223, 80224, 80225, 80226, 80227, 80228, 80229, 80230, 80231, 80232, 80234, 80235, 80236, 80237, 80238, 80239, 8024, 8025, 8026, 8027, 8028, 8029, 80300, 80301, 80302, 80306, 80311, 80312, 80313, 80315, 80316, 80320, 80321, 80322, 80324, 80325, 80326, 80331, 80332, 80336, 80341, 80350, 80360, 80362, 80366, 80370, 80372, 80375, 80376, 80400, 80410, 80412, 80416, 80420, 80421, 80422, 80425, 80426, 80430, 80431, 80432, 80435, 80436, 80441, 80466, 80470, 80473, 80475, 80476, 80485, 80500, 80501, 80502, 80503, 80504, 80505, 80506, 80507, 80508, 8052, 8053, 8054, 8055, 8056, 8057, 8058, 80600, 80601, 80602, 80603, 80604, 80605, 80606, 80607, 80608, 80609, 80610, 80613, 80616, 80620, 80621, 80622, 80623, 80624, 80625, 80626, 80629, 80631, 80639, 8064, 8065, 80660, 80662, 8068, 80700, 80701, 80702, 80703, 80704, 80705, 80706, 80707, 80708, 80709, 80710, 80711, 80713, 80718, 80719, 8072, 8073, 8074, 8075, 8076, 8080, 8081, 8082, 8083, 80841, 80842, 80843, 80844, 80849, 80851, 80853, 8088, 8089, 81000, 81001, 81002, 81003, 81010, 81011, 81012, 81013, 81100, 81101, 81102, 81103, 81109, 81110, 81112, 81200, 81201, 81202, 81203, 81209, 81210, 81212, 81219, 81220, 81221, 81230, 81231, 81240, 81241, 81242, 81243, 81244, 81249, 81250, 81251, 81252, 81254, 81259, 81301, 81302, 81303, 81304, 81305, 81306, 81307, 81308, 81311, 81312, 81313, 81314, 81315, 81318, 81321, 81322, 81323, 81331, 81332, 81333, 81341, 81342, 81343, 81344, 81351, 81352, 81353, 81354, 81380, 81381, 81382, 81383, 81391, 81392, 81393, 81400, 81401, 81402, 81403, 81404, 81405, 81406, 81407, 81408, 81409, 81410, 81411, 81412, 81413, 81415, 81416, 81418, 81419, 81500, 81501, 81502, 81503, 81504, 81509, 81510, 81511, 81512, 81513, 81514, 81519, 81600, 81601, 81602, 81603, 81610, 81611, 81612, 81613, 8170, 8171, 8190, 8191, 82000, 82001, 82002, 82003, 82009, 82019, 82020, 82021, 82022, 82030, 82031, 82032, 8208, 8209, 82100, 82101, 82110, 82111, 82120, 82121, 82122, 82123, 82129, 82130, 82131, 82132, 82133, 82139, 8220, 8221, 82300, 82301, 82302, 82310, 82311, 82312, 82320, 82321, 82322, 82330, 82331, 82332, 82342, 82380, 82381, 82382, 82390, 82391, 82392, 8240, 8241, 8242, 8243, 8244, 8245, 8246, 8247, 8248, 8249, 8250, 8251, 82520, 82521, 82522, 82523, 82524, 82525, 82529, 82530, 82531, 82532, 82533, 82534, 82535, 82539, 8260, 8261, 8270, 8271, 8280, 8290, 8300, 83100, 83101, 83102, 83103, 83104, 83109, 83114, 83200, 83201, 83202, 83209, 83210, 83213, 8322, 83300, 83301, 83302, 83303, 83304, 83305, 83309, 83310, 83311, 83314, 83315, 83400, 83401, 83402, 83410, 83411, 83412, 83500, 83501, 83503, 8360, 8361, 8362, 8363, 8364, 83650, 83651, 83652, 83659, 83661, 8370, 8371, 83801, 83802, 83803, 83804, 83805, 83806, 83809, 83810, 83812, 83813, 83815, 83816, 83819, 83900, 83901, 83902, 83903, 83904, 83905, 83906, 83907, 83908, 83920, 83921, 83940, 83941, 83942, 83961, 83969, 83979, 8400, 8401, 8403, 8404, 8405, 8406, 8407, 8408, 8409, 8410, 8411, 8412, 8413, 8418, 8419, 84200, 84201, 84209, 84210, 84212, 84213, 84219, 8430, 8438, 8439, 8440, 8441, 8442, 8448, 8449, 84500, 84502, 84503, 84509, 84510, 84511, 84512, 84519, 8460, 8461, 8468, 8469, 8470, 8471, 8472, 8473, 8479, 8483, 84842, 84849, 8488, 8489, 8500, 8501, 85011, 8502, 8504, 8505, 8509, 85100, 85101, 85102, 85103, 85105, 85106, 85109, 85110, 85120, 85121, 85125, 85131, 85135, 85140, 85141, 85142, 85145, 85146, 85160, 85162, 85173, 85175, 85176, 85180, 85181, 85182, 85183, 85184, 85185, 85186, 85189, 85190, 85191, 85196, 85200, 85201, 85202, 85203, 85204, 85205, 85206, 85209, 85210, 85211, 85212, 85215, 85216, 85219, 85220, 85221, 85222, 85223, 85224, 85225, 85226, 85229, 85230, 85231, 85232, 85235, 85236, 85239, 85240, 85241, 85242, 85245, 85246, 85256, 85300, 85301, 85302, 85304, 85305, 85306, 85309, 85310, 85312, 85314, 85400, 85401, 85402, 85403, 85404, 85405, 85406, 8600, 8601, 8602, 8603, 8604, 8605, 86100, 86101, 86102, 86103, 86110, 86112, 86113, 86120, 86121, 86122, 86130, 86131, 86132, 8620, 8621, 86221, 86222, 86229, 86232, 86239, 8628, 8629, 8630, 8631, 86320, 86321, 86329, 86330, 86331, 86339, 86340, 86341, 86342, 86343, 86344, 86345, 86346, 86349, 86350, 86351, 86352, 86353, 86354, 86355, 86356, 86380, 86381, 86382, 86383, 86384, 86389, 86392, 86393, 86394, 86399, 86400, 86401, 86402, 86403, 86404, 86405, 86409, 86410, 86411, 86412, 86413, 86414, 86415, 86419, 86500, 86501, 86502, 86503, 86504, 86509, 86510, 86511, 86512, 86513, 86514, 86600, 86601, 86602, 86603, 86610, 86611, 86612, 86613, 8670, 8671, 8672, 8674, 8676, 8677, 8678, 8679, 86800, 86801, 86802, 86803, 86804, 86809, 86810, 86811, 86812, 86813, 86814, 86819, 8690, 8691, 8700, 8701, 8702, 8703, 8704, 8708, 8709, 8710, 8711, 8712, 8713, 8714, 8715, 8716, 8719, 87200, 87201, 87202, 87210, 87211, 87261, 87264, 87269, 87271, 87279, 8728, 8730, 8731, 87320, 87321, 87322, 87323, 87330, 87332, 87333, 87340, 87341, 87342, 87343, 87344, 87349, 87350, 87351, 87352, 87353, 87354, 87359, 87360, 87361, 87362, 87363, 87364, 87365, 87369, 87371, 87373, 87374, 87379, 8738, 8739, 87401, 87402, 87411, 87412, 8742, 8744, 8745, 8748, 8749, 8750, 8751, 8760, 8761, 8770, 8771, 8780, 8782, 8783, 8784, 8786, 8790, 8791, 8792, 8793, 8794, 8795, 8796, 8797, 8798, 8799, 88000, 88001, 88002, 88003, 88009, 88010, 88013, 88019, 88020, 88023, 88029, 88100, 88101, 88102, 88110, 88111, 88112, 88120, 88121, 88122, 8820, 8821, 8822, 8830, 8831, 8832, 8840, 8841, 8842, 8850, 8851, 8860, 8861, 8870, 8871, 8872, 8873, 8875, 8900, 8901, 8902, 8910, 8911, 8912, 8920, 8921, 8922, 8930, 8931, 8932, 8940, 8941, 8950, 8960, 8961, 8970, 8971, 8972, 8973, 8977, 90000, 90001, 90002, 90003, 9001, 90081, 90082, 90089, 9009, 9010, 9011, 9012, 9013, 90140, 90141, 90142, 90181, 90182, 9019, 9020, 90210, 90211, 90220, 90221, 90222, 90223, 90225, 90226, 90229, 90233, 90234, 90241, 90242, 90249, 90251, 90253, 90254, 90287, 90289, 9029, 90301, 90302, 9031, 9032, 9033, 9034, 9035, 9038, 9039, 9040, 9041, 9042, 9043, 90441, 90442, 90451, 90452, 90453, 9046, 9047, 9048, 9049, 9050, 9051, 9052, 9053, 9054, 9055, 9056, 9057, 9058, 9059, 9060, 9061, 9062, 9063, 9064, 9065, 9066, 9067, 9068, 9069, 9070, 9071, 9072, 9073, 9074, 9075, 9079, 9080, 9081, 9082, 9083, 9084, 9086, 9089, 9090, 9091, 9092, 9093, 9094, 9095, 9099, 9100, 9101, 9102, 9103, 9104, 9106, 9108, 9110, 9112, 9114, 9115, 9116, 9117, 9118, 9120, 9122, 9124, 9125, 9128, 9130, 9131, 9132, 9134, 9135, 9136, 9137, 9138, 9140, 9141, 9142, 9143, 9144, 9145, 9147, 9149, 9150, 9151, 9152, 9153, 9155, 9156, 9157, 9158, 9159, 9160, 9161, 9162, 9163, 9164, 9165, 9167, 9168, 9169, 9170, 9171, 9172, 9173, 9175, 9176, 9177, 9178, 9180, 9181, 9182, 9189, 9190, 9191, 9192, 9194, 9195, 9196, 9198, 9199, 920, 9210, 9211, 9212, 9213, 9219, 9220, 9221, 9222, 92231, 92232, 92233, 9224, 9228, 9229, 92300, 92301, 92302, 92303, 92309, 92310, 92311, 92320, 92321, 9233, 9238, 9239, 92400, 92401, 92410, 92411, 92420, 92421, 9243, 9244, 9245, 9248, 9249, 9251, 9252, 9260, 92611, 92612, 92619, 9268, 92701, 92710, 92720, 92721, 9273, 9278, 92800, 92801, 92810, 92811, 92820, 92821, 9283, 9289, 9290, 9300, 9301, 9308, 9309, 931, 932, 9330, 9331, 9340, 9341, 9348, 9349, 9350, 9351, 9352, 936, 937, 938, 9390, 9392, 9393, 94100, 94103, 94108, 94111, 94116, 94120, 94126, 94127, 94128, 94201, 94203, 94204, 94205, 94209, 94212, 94213, 94214, 94215, 94221, 94222, 94223, 94224, 94229, 94231, 94232, 94233, 94234, 94239, 94301, 94302, 94311, 94320, 94321, 94325, 94331, 94332, 94333, 94335, 94339, 94400, 94401, 94402, 94403, 94407, 94410, 94411, 94416, 94420, 94421, 94422, 94423, 94425, 94426, 94428, 94431, 94432, 94436, 94500, 94502, 94503, 94504, 94506, 94510, 94512, 94514, 94516, 94519, 94522, 94523, 94524, 94526, 94530, 94532, 94534, 9462, 9470, 9471, 9472, 9473, 9478, 94800, 94810, 94811, 94820, 94840, 94850, 94890, 9490, 9500, 9501, 9503, 9509, 9510, 9511, 9512, 9513, 9514, 9515, 9517, 9518, 9519, 95200, 95201, 95203, 95204, 95205, 95206, 95207, 95208, 95209, 95210, 95211, 95214, 95215, 95217, 95219, 9522, 9523, 9528, 9529, 9530, 9531, 9533, 9534, 9535, 9539, 9540, 9550, 9551, 9552, 9553, 9554, 9555, 9556, 9557, 9558, 9559, 9560, 9561, 9562, 9563, 9564, 9565, 9569, 9570, 9571, 9578, 9579, 9580, 9581, 9582, 9583, 9584, 9585, 9586, 9587, 9588, 95891, 95892, 95893, 95899, 95901, 95909, 95911, 95912, 95913, 95914, 95919, 9592, 9593, 9594, 9595, 9596, 9597, 9598, 9599

ICD-10 codes:

S0000XD, S0001XA, S0003XA, S0003XD, S0005XA, S0006XA, S0010XA, S0011XA, S0011XD, S0012XA, S0012XD, S00201A, S00202A, S00211A, S00212A, S00219A, S0030XA, S0031XA, S0033XA, S00411A, S00412A, S00431A, S00432A, S00462A, S00511A, S00512A, S00522A, S00531A, S00532A, S00572A, S0080XA, S0081XA, S0081XD, S0083XA, S0083XD, S0086XA, S0090XA, S0091XA, S0093XA, S0100XA, S0100XD, S0100XS, S0101XA, S0101XD, S0101XS, S0102XA, S0103XA, S0104XA, S01111A, S01112A, S01119A, S01121A, S01122A, S01131S, S01132S, S01152A, S0121XA, S0121XD, S0122XA, S01302A, S01311A, S01312A, S01319A, S01321A, S01351A, S01401A, S01402A, S01411A, S01412A, S01419A, S01422A, S01431A, S01432A, S01451A, S01452A, S01452D, S01511A, S01511D, S01512A, S01512D, S01521A, S01532A, S01551A, S01552A, S0180XA, S0180XD, S0180XS, S0181XA, S0181XD, S0182XA, S0183XA, S0184XA, S0185XA, S0190XS, S0191XA, S0191XD, S020XXA, S020XXB, S020XXD, S02101A, S02102A, S02102D, S02109A, S0210XA, S02113A, S02113D, S02118A, S02118B, S02118G, S02119A, S02119B, S02119D, S02119S, S0211CA, S0211GA, S0211GB, S0211GD, S0211HA, S0211HB, S0211HD, S0219XA, S0219XB, S0219XD, S0219XS, S022XXA, S022XXB, S022XXD, S0231XA, S0231XB, S0231XD, S0231XG, S0232XA, S0232XD, S023XXA, S023XXD, S023XXS, S02400A, S02401A, S02401B, S02401D, S02402A, S02402B, S02402D, S0240CA, S0240CB, S0240CD, S0240DA, S0240DD, S0240EA, S0240ED, S0240ES, S0240FA, S0240FB, S0240FD, S0240FS, S02411A, S02412A, S02413A, S0242XA, S0242XB, S025XXA, S025XXB, S025XXD, S02600A, S02600B, S02601A, S02601B, S02601K, S02602A, S02602B, S02602D, S02609A, S02609B, S02609D, S02609K, S02610A, S02611A, S02612A, S0261XA, S0261XB, S02621A, S02621B, S02622A, S02622B, S0262XA, S0262XB, S02631A, S02632A, S0263XA, S02641A, S02641B, S02642A, S02642B, S0264XA, S0264XB, S02651A, S02651B, S02652A, S02652B, S0265XA, S0265XB, S0265XK, S0266XA, S0266XB, S02670A, S02671A, S02672B, S0267XA, S0269XA, S0280XA, S0280XD, S0280XS, S0281XA, S0281XD, S0282XA, S0282XD, S02831A, S02832A, S02841A, S0285XA, S028XXA, S028XXB, S028XXD, S0291XA, S0291XD, S0291XS, S0292XA, S0292XB, S0292XD, S0300XA, S0302XA, S0303XA, S030XXA, S032XXA, S038XXA, S04011A, S04012A, S0421XS, S0450XA, S0451XA, S0452XA, S0462XS, S04892A, S0500XA, S0501XA, S0502XA, S0510XA, S0511XA, S0511XD, S0512XA, S0512XD, S0521XA, S0522XA, S0531XA, S0531XD, S0532XA, S0541XA, S0541XS, S0542XA, S0542XD, S0571XA, S0571XS, S0572XA, S058X1A, S058X1S, S058X2A, S058X2D, S058X2S, S058X9A, S0591XA, S0591XS, S0592XA, S0592XD, S0592XS, S060X0A, S060X0S, S060X1A, S060X9A, S060X9D, S060X9S, S061X0A, S061X0D, S061X1A, S061X6A, S061X7A, S061X8A, S061X9A, S061X9D, S062X0A, S062X0D, S062X1A, S062X6A, S062X7A, S062X9A, S062X9D, S062X9S, S06300A, S06300D, S06300S, S06301A, S06309A, S06309S, S06310A, S06311A, S06319A, S06320A, S06321A, S06329A, S06330A, S06339A, S06340A, S06340S, S06341A, S06347A, S06349A, S06350A, S06351A, S06357A, S06359A, S06359S, S06360A, S06360S, S06361A, S06362A, S06366A, S06369A, S06369S, S06370A, S06370S, S06371A, S06377A, S06379A, S06379D, S06380D, S064X0A, S064X0S, S064X1A, S064X2A, S064X6A, S064X7A, S064X9A, S064X9D, S064X9S, S065X0A, S065X0D, S065X0S, S065X1A, S065X1D, S065X3A, S065X5S, S065X6A, S065X7A, S065X8A, S065X9A, S065X9D, S065X9S, S066X0A, S066X0D, S066X0S, S066X1A, S066X1D, S066X3A, S066X6A, S066X7A, S066X8A, S066X9A, S066X9D, S066X9S, S06810A, S06810S, S06819A, S06829A, S06890A, S06890S, S06891A, S06897A, S06899A, S06899S, S069X0A, S069X0D, S069X0S, S069X1A, S069X1D, S069X1S, S069X3A, S069X5S, S069X7A, S069X9A, S069X9D, S069X9S, S080XXA, S090XXA, S0911XA, S0912XA, S0921XA, S0921XD, S0922XA, S09391A, S098XXA, S098XXS, S0990XA, S0990XS, S0991XA, S0992XA, S0993XA, S0993XS, S100XXA, S1011XA, S1080XA, S1081XA, S1083XA, S1085XA, S1091XA, S1093XA, S1096XA, S11011A, S11013A, S11021A, S11023A, S1111XA, S1113XA, S1121XA, S1122XA, S1123XA, S1181XA, S1182XA, S1183XA, S1184XA, S1189XA, S1190XA, S1191XA, S1191XD, S1194XA, S12000A, S12000D, S12000G, S12000S, S12001A, S12001D, S1201XA, S1201XD, S1202XA, S1202XD, S1202XK, S12030A, S12031A, S12040A, S12041A, S12090A, S12091A, S12100A, S12100D, S12100G, S12100K, S12100S, S12101A, S12110A, S12110D, S12110G, S12110S, S12111A, S12111D, S12112A, S12112S, S12120A, S12120D, S12121A, S12130A, S12190A, S12191A, S12200A, S12200D, S12200S, S12201A, S12201D, S12290A, S12291A, S12300A, S12300D, S12300S, S12301A, S12330A, S12390A, S12390D, S12391A, S12400A, S12400D, S12400S, S12401A, S12430A, S12490A, S12491A, S12500A, S12500D, S12500S, S12501A, S12530A, S12531A, S12550A, S12590A, S12590D, S12591A, S12600A, S12600D, S12600S, S12601A, S12601D, S12630A, S12690A, S12690D, S12691A, S128XXA, S129XXA, S129XXD, S129XXS, S130XXA, S13100A, S13120A, S13121A, S13130A, S13131A, S13140A, S13141A, S13150A, S13151A, S13160A, S13161A, S13171A, S13181A, S134XXA, S134XXD, S138XXA, S139XXA, S140XXA, S14101A, S14102A, S14102S, S14103A, S14103S, S14104A, S14104S, S14105A, S14105S, S14106A, S14106S, S14107A, S14107S, S14109A, S14109D, S14109S, S14111A, S14112A, S14113A, S14114A, S14115A, S14116A, S14117A, S14122A, S14123A, S14123S, S14124A, S14125A, S14126A, S14127A, S14129A, S14129D, S14129S, S14132A, S14135A, S14152A, S14153A, S14153S, S14154A, S14155A, S14155S, S14156A, S14156D, S14156S, S14157A, S14158A, S14159D, S14159S, S142XXA, S143XXA, S143XXD, S143XXS, S144XXA, S145XXA, S145XXD, S15001S, S15021A, S15091A, S15092A, S15101A, S15109A, S15191A, S15192A, S15211A, S15212A, S15391A, S15392A, S158XXA, S159XXA, S161XXA, S162XXA, S168XXA, S178XXA, S178XXD, S1980XA, S1982XA, S1983XA, S1985XA, S1989XA, S199XXA, S199XXS, S2001XA, S2002XA, S20111A, S20112A, S20121A, S20122A, S2020XA, S20211A, S20212A, S20219A, S20219S, S20221A, S20222A, S20229A, S20311A, S20312A, S20319A, S20319D, S20321A, S20322A, S20329A, S20361D, S20369A, S20402A, S20411A, S20412A, S20419A, S20461A, S20469A, S21011A, S21012A, S21101A, S21102A, S21111A, S21112A, S21119A, S21119D, S21132A, S21139A, S21141A, S21209S, S21211A, S21212A, S21219D, S21311A, S21312A, S21331A, S21332A, S21341A, S21411A, S21412A, S2190XA, S22000A, S22009A, S22009D, S22010A, S22011A, S22018A, S22019A, S22019D, S22019S, S22020A, S22021A, S22028A, S22029A, S22029D, S22030A, S22031A, S22032A, S22038A, S22039A, S22039D, S22040A, S22041A, S22041G, S22042A, S22048A, S22049A, S22049D, S22050A, S22051A, S22051D, S22052A, S22058A, S22059A, S22059D, S22059G, S22060A, S22060S, S22061A, S22061D, S22061S, S22062A, S22068A, S22069A, S22069D, S22069G, S22069S, S22070A, S22071A, S22072A, S22078A, S22079A, S22079D, S22079G, S22080A, S22080D, S22080S, S22081A, S22081D, S22081S, S22082A, S22088A, S22088S, S22089A, S22089D, S22089G, S22089S, S2220XA, S2220XD, S2220XK, S2220XS, S2221XA, S2221XD, S2221XS, S2222XA, S2222XD, S2224XA, S2231XA, S2231XB, S2231XD, S2231XK, S2231XS, S2232XA, S2232XD, S2232XG, S2232XS, S2239XA, S2239XD, S2239XG, S2241XA, S2241XD, S2241XG, S2241XS, S2242XA, S2242XD, S2242XG, S2243XA, S2243XD, S2243XG, S2243XS, S2249XA, S2249XD, S2249XG, S2249XS, S225XXA, S225XXD, S23162A, S2329XA, S2329XS, S233XXA, S2341XA, S239XXA, S240XXA, S24101A, S24102A, S24102D, S24102S, S24103A, S24103D, S24103S, S24104A, S24104D, S24104S, S24109A, S24109S, S24112A, S24112S, S24113S, S24114A, S24151S, S24152A, S24152S, S24153A, S24153S, S24154A, S2500XS, S2501XA, S2502XA, S2509XA, S25101A, S25191A, S25491A, S25499A, S25501A, S25502A, S25512A, S2600XA, S2601XA, S26020A, S26021A, S2609XA, S2610XA, S2611XA, S2612XA, S2619XA, S2690XA, S2691XA, S2699XA, S270XXA, S271XXA, S271XXD, S272XXA, S272XXD, S27301A, S27309A, S27321A, S27321D, S27321S, S27322A, S27329A, S27329D, S27331A, S27331S, S27339A, S27391A, S27391S, S27409A, S27431A, S27439A, S27491A, S2752XA, S2753XA, S2769XA, S27802A, S27803A, S27808A, S27809A, S27813A, S27818A, S27819A, S27819D, S27892A, S27892D, S280XXA, S29011A, S29012A, S29092A, S298XXA, S298XXS, S299XXA, S300XXA, S300XXD, S300XXS, S301XXA, S301XXD, S3021XA, S3022XA, S3023XA, S303XXA, S30810A, S30811A, S30812A, S30813A, S30814A, S30814D, S30817A, S30817D, S30820A, S30821A, S30860A, S30861A, S3091XA, S3092XA, S3095XA, S3098XA, S31000A, S31001A, S31010A, S31031A, S31041A, S31100A, S31101A, S31103A, S31103D, S31104A, S31104S, S31105D, S31109A, S31110A, S31110D, S31111A, S31112A, S31113A, S31114A, S31119A, S31119D, S31129A, S31132A, S31133A, S31134A, S31135A, S31139A, S31143A, S31144A, S31149A, S3121XA, S3130XA, S3131XA, S3141XA, S31502A, S31511A, S31602A, S31603A, S31609A, S31611A, S31612A, S31613A, S31614A, S31615A, S31619A, S31620A, S31624A, S31630A, S31631A, S31633A, S31634A, S31639A, S31640A, S31641A, S31642A, S31643A, S31644A, S31645A, S31649A, S31801A, S31809A, S31811A, S31814A, S31819A, S31821A, S31823A, S31824A, S31829A, S31829D, S31831A, S32000A, S32001A, S32001D, S32009A, S32009D, S32009S, S32010A, S32010D, S32010S, S32011A, S32011D, S32011G, S32012A, S32018A, S32018B, S32018D, S32019A, S32019D, S32019G, S32019S, S32020A, S32021A, S32021D, S32021K, S32021S, S32022A, S32022B, S32028A, S32028D, S32029A, S32029D, S32029G, S32030A, S32030D, S32031A, S32031D, S32032A, S32032K, S32038A, S32039A, S32039D, S32039G, S32040A, S32040D, S32041A, S32041D, S32041G, S32042A, S32048A, S32049A, S32049B, S32049D, S32049S, S32050A, S32051A, S32052A, S32058A, S32058D, S32059A, S32059D, S32059K, S3210XA, S3210XB, S3210XD, S3210XG, S3210XK, S3210XS, S32110A, S32111A, S32119A, S32119D, S32120A, S32121A, S32129A, S32129B, S32139A, S3214XA, S3215XA, S3219XA, S3219XD, S322XXA, S322XXG, S32301A, S32301B, S32301D, S32302A, S32302D, S32311A, S32312A, S32391A, S32391B, S32392A, S32392B, S32392D, S32399D, S32401A, S32401D, S32401S, S32402A, S32402D, S32402S, S32411A, S32412A, S32414A, S32415A, S32421A, S32421S, S32422A, S32422D, S32424A, S32425A, S32431A, S32431K, S32432A, S32432K, S32433A, S32434A, S32435A, S32441A, S32442A, S32442K, S32445A, S32451A, S32452A, S32454A, S32455A, S32461A, S32462A, S32464A, S32465A, S32471A, S32472A, S32475A, S32481A, S32482A, S32491A, S32492A, S32492D, S32501A, S32501D, S32502A, S32502D, S32509A, S32511A, S32511D, S32512A, S32512D, S32519A, S32591A, S32591D, S32592A, S32592D, S32599A, S32599S, S32601A, S32602A, S32691A, S32692A, S32810A, S32810B, S32810D, S32811A, S32811B, S3282XA, S3282XD, S3289XA, S3289XD, S329XXA, S329XXD, S329XXG, S33101A, S33111A, S33131A, S33141A, S332XXA, S3339XA, S334XXA, S335XXA, S336XXA, S338XXA, S339XXA, S3401XA, S34101A, S34102A, S34104A, S34104D, S34105A, S34109A, S34121A, S34139A, S3421XA, S3422XA, S343XXA, S344XXA, S344XXS, S345XXA, S348XXS, S349XXA, S3509XA, S3510XA, S3519XA, S35228A, S35291A, S35292A, S35298A, S35318A, S35319A, S35321A, S35511A, S35512A, S35515A, S35534A, S358X1A, S358X8A, S3591XA, S3599XA, S3600XD, S36020A, S36021A, S36021D, S36029A, S36029D, S36030A, S36031A, S36031D, S36031S, S36032A, S36032D, S36039A, S36039S, S3609XA, S36112A, S36113A, S36113D, S36114A, S36115A, S36116A, S36118A, S36118D, S36119A, S36123A, S36128A, S3613XA, S3613XS, S36220A, S36222A, S36230A, S36239A, S36242A, S36261A, S36262A, S3630XA, S3632XA, S3633XA, S3639XA, S36409A, S36419A, S36420A, S36428A, S36430A, S36438A, S36439A, S36490A, S36498A, S36513A, S36520A, S36522A, S36523A, S36528A, S36530A, S36531A, S36532A, S36533A, S36538A, S36539A, S36590A, S36591A, S36593A, S36598A, S36599S, S3662XA, S3663XA, S3669XA, S3681XA, S36892A, S36892D, S36893A, S36898A, S36898S, S36899A, S37001A, S37002A, S37009A, S37011A, S37012A, S37012D, S37019A, S37022A, S37031A, S37032A, S37041A, S37042A, S37051A, S37052A, S37061A, S37062A, S37091A, S3710XA, S3713XA, S3719XA, S3720XA, S3722XA, S3723XA, S3729XA, S3730XA, S3733XA, S3739XA, S3763XA, S3769XA, S37812A, S37813A, S37818A, S37828A, S37828D, S37829A, S37892A, S3790XA, S3792XA, S3792XD, S381XXA, S381XXS, S38221A, S39011A, S39011D, S39012A, S39012D, S39013A, S39021A, S39092A, S39093A, S3981XA, S3982XA, S39840A, S39848A, S3991XA, S3992XA, S40011A, S40012A, S40012D, S40019A, S40021A, S40021D, S40021S, S40022A, S40029A, S40211A, S40211D, S40212A, S40221A, S40811A, S40812A, S40819A, S40819D, S40822A, S40852A, S40861A, S40861D, S40872A, S40911A, S40921A, S40922A, S41001A, S41002A, S41002D, S41011A, S41012A, S41041A, S41042A, S41101A, S41102A, S41111A, S41111D, S41112A, S41112D, S41132A, S41141A, S41142A, S41151A, S41152A, S42001A, S42001D, S42001K, S42002A, S42002D, S42002K, S42009A, S42011A, S42012A, S42012B, S42018A, S42021A, S42021D, S42022A, S42022D, S42024A, S42025A, S42031A, S42031B, S42032A, S42032B, S42032D, S42034A, S42035A, S42101A, S42101D, S42102A, S42102D, S42109A, S42109D, S42111A, S42112A, S42114A, S42115A, S42115B, S42121A, S42121S, S42122A, S42124A, S42125A, S42131A, S42132A, S42134A, S42135A, S42141A, S42141D, S42142A, S42144A, S42145A, S42151A, S42152A, S42191A, S42192A, S42201A, S42201B, S42201D, S42201K, S42201P, S42201S, S42202A, S42202B, S42202D, S42202K, S42202S, S42211A, S42211D, S42212A, S42212B, S42212D, S42212K, S42212S, S42214A, S42214D, S42215A, S42215D, S42221A, S42222A, S42222D, S42231A, S42232A, S42251A, S42252A, S42252D, S42252P, S42254A, S42255A, S42261A, S42262A, S42291A, S42291D, S42291G, S42291S, S42292A, S42292B, S42292D, S42292K, S42294A, S42295A, S42295D, S42301A, S42301B, S42301D, S42301G, S42301K, S42301P, S42301S, S42302A, S42302D, S42302K, S42302P, S42321B, S42322A, S42324A, S42331A, S42331B, S42332A, S42332B, S42335A, S42341A, S42342A, S42342K, S42351A, S42351B, S42351K, S42352A, S42352B, S42391A, S42391B, S42391K, S42392A, S42392P, S42401A, S42401B, S42401D, S42401K, S42402A, S42402B, S42402D, S42402K, S42402S, S42411A, S42411B, S42412A, S42412B, S42415A, S42421A, S42421B, S42422A, S42422B, S42431A, S42432A, S42435A, S42442A, S42445A, S42451A, S42451B, S42451D, S42452A, S42452P, S42461B, S42462A, S42462P, S42472K, S42491A, S42491B, S42491K, S42492A, S42492B, S42494A, S4291XA, S4291XD, S4292XA, S4292XD, S4292XG, S43001A, S43004A, S43004D, S43005A, S43014A, S43015A, S43024A, S43031A, S43035A, S43081A, S43082A, S43084A, S43101A, S43102A, S43111A, S43112A, S43121A, S43224A, S43225A, S43312A, S43401A, S43402A, S43409A, S43421A, S43422A, S43431A, S43432A, S43439A, S43491A, S43492A, S4351XA, S4401XA, S4402XA, S4411XA, S4421XA, S4422XA, S4432XA, S4442XA, S4491XD, S4492XA, S4492XD, S45001A, S45091A, S45092A, S45102D, S45111A, S45112A, S45192A, S45199S, S45211A, S45301A, S45811A, S45812A, S45911A, S46001A, S46001D, S46002A, S46009D, S46011A, S46011D, S46011S, S46012A, S46012D, S46012S, S46021D, S46022A, S46091A, S46111A, S46112A, S46121A, S46122A, S46211A, S46211S, S46212A, S46221A, S46311A, S46321A, S46811A, S46812A, S46821A, S46822A, S46911A, S46911D, S46912A, S471XXA, S48922A, S49191D, S49192A, S4982XA, S4990XA, S4991XA, S4991XS, S4992XA, S4992XS, S5001XA, S5002XA, S5011XA, S5012XA, S50311A, S50311D, S50312A, S50319A, S50362A, S50811A, S50811D, S50812A, S50819A, S50821A, S50822A, S50852A, S50861A, S50871A, S50872A, S50911A, S51001A, S51002A, S51011A, S51011D, S51012A, S51012D, S51022A, S51031A, S51032A, S51801A, S51802A, S51811A, S51811D, S51812A, S51819A, S51821A, S51822A, S51831A, S51832A, S51832D, S51841A, S51842A, S51851A, S51852A, S51852S, S52001A, S52001B, S52002A, S52021A, S52021B, S52021D, S52022A, S52022B, S52022G, S52024A, S52024B, S52025A, S52025D, S52031A, S52031B, S52032A, S52041A, S52042A, S52042B, S52045A, S52091A, S52092A, S52092S, S52101A, S52102B, S52102D, S52121A, S52121B, S52122A, S52122B, S52124A, S52125A, S52125D, S52131A, S52131B, S52132A, S52132K, S52134A, S52135A, S52182B, S52201A, S52201B, S52201D, S52201S, S52202A, S52202B, S52202D, S52202K, S52202M, S52221A, S52222A, S52222B, S52224A, S52225A, S52234A, S52251A, S52251B, S52252A, S52252B, S52252D, S52254A, S52255B, S52271A, S52271B, S52272A, S52272B, S52292A, S52292B, S52301A, S52301B, S52302A, S52302B, S52302M, S52302P, S52321A, S52322A, S52322B, S52332A, S52335A, S52341A, S52351A, S52351B, S52352A, S52352B, S52352D, S52371A, S52371B, S52372A, S52372B, S52391A, S52391B, S52392B, S52501A, S52501B, S52501D, S52501E, S52501G, S52501K, S52501P, S52501S, S52502A, S52502B, S52502D, S52502K, S52502M, S52502P, S52511A, S52511B, S52512A, S52512B, S52512D, S52514A, S52515A, S52531A, S52531D, S52551A, S52552A, S52571A, S52571B, S52571D, S52572A, S52572B, S52572D, S52591A, S52591B, S52591D, S52592A, S52592B, S52592D, S52601A, S52601B, S52601M, S52601P, S52602A, S52602B, S52611A, S52611B, S52612A, S52612B, S52612D, S52614A, S52615A, S52691A, S52691B, S52692A, S52692B, S5291XA, S5291XB, S5291XD, S5292XA, S5292XD, S5292XS, S53005A, S53095A, S53102A, S53104A, S53105A, S53115A, S53121A, S53124A, S53125A, S53144A, S53145A, S53195A, S5321XA, S5322XA, S5331XA, S5332XA, S53401A, S53402A, S53442A, S53491A, S53492A, S5401XA, S5402XA, S5411XA, S5411XS, S5412XA, S5412XS, S5421XA, S5422XA, S5431XA, S548X1S, S55012A, S55111A, S55112A, S55191A, S55192A, S55211A, S55212A, S55292A, S55812A, S55901A, S55912A, S56021A, S56114A, S56116A, S56118A, S56121A, S56122A, S56123A, S56124A, S56125A, S56126A, S56127A, S56221A, S56222A, S56322A, S56421A, S56426A, S56502A, S56521A, S56522A, S56821A, S56822A, S5701XS, S5702XA, S5781XA, S5782XA, S5782XS, S59001A, S59092A, S59801A, S59802A, S59811A, S59901A, S59902A, S59909A, S6000XA, S60011A, S60012A, S60021A, S60041A, S60052A, S60111A, S60112A, S60131A, S60211A, S60212A, S60221A, S60222A, S60229A, S60311A, S60322A, S60352A, S60362A, S60372A, S60410A, S60411A, S60412A, S60413A, S60414A, S60416A, S60417A, S60419A, S60422A, S60428A, S60451A, S60454A, S60467A, S60470A, S60472A, S60474A, S60511A, S60512A, S60519A, S60521A, S60522A, S60551A, S60561A, S60562A, S60571A, S60572A, S60811A, S60812A, S60862A, S60942A, S60943A, S60944A, S61001A, S61002A, S61011A, S61011D, S61012A, S61012D, S61012S, S61031A, S61051A, S61052A, S61102A, S61111A, S61151A, S61200A, S61202A, S61204A, S61205A, S61207A, S61210A, S61211A, S61211D, S61212A, S61213A, S61213D, S61214A, S61215A, S61216A, S61217A, S61218A, S61219A, S61223A, S61225A, S61227A, S61231A, S61231S, S61232A, S61233A, S61236A, S61240A, S61245A, S61250A, S61251A, S61251D, S61252A, S61253A, S61254A, S61256A, S61257A, S61301A, S61303A, S61304A, S61307A, S61310A, S61311A, S61312A, S61313A, S61315A, S61317A, S61401A, S61402A, S61409A, S61411A, S61412A, S61419A, S61421A, S61422A, S61431A, S61432A, S61432D, S61441A, S61442A, S61451A, S61452A, S61452D, S61459A, S61502A, S61511A, S61511D, S61512A, S61512D, S61519A, S61522A, S61531A, S61532A, S61551A, S61552A, S62001A, S62002A, S62002B, S62002K, S62012A, S62015A, S62021A, S62022A, S62032A, S62035A, S62101A, S62101B, S62101D, S62102A, S62102D, S62111A, S62112A, S62114A, S62121A, S62122A, S62141A, S62142A, S62152A, S62161D, S62201A, S62202A, S62211A, S62221A, S62221B, S62222A, S62231A, S62232A, S62232B, S62232P, S62235A, S62241A, S62241B, S62291B, S62300A, S62301B, S62302A, S62302B, S62303A, S62303B, S62304B, S62304K, S62305B, S62306A, S62306B, S62306D, S62307A, S62307B, S62308D, S62309A, S62309B, S62310A, S62311A, S62311B, S62312A, S62313A, S62313B, S62315A, S62315B, S62316A, S62316D, S62317A, S62317B, S62320B, S62321A, S62322A, S62323A, S62323B, S62324A, S62324B, S62325A, S62325B, S62326A, S62326B, S62327A, S62327B, S62330B, S62331A, S62332B, S62333A, S62335A, S62336A, S62337A, S62341A, S62342A, S62346A, S62352B, S62354A, S62354B, S62356A, S62357A, S62366A, S62391A, S62392A, S62392B, S62393A, S62394A, S62395A, S62396A, S62397A, S62397B, S62398A, S62501A, S62501B, S62502A, S62511A, S62511B, S62512A, S62512B, S62514A, S62514B, S62514D, S62515A, S62521A, S62521B, S62522A, S62522B, S62525A, S62601A, S62602D, S62603A, S62603B, S62604A, S62605A, S62605S, S62606A, S62607A, S62607D, S62609A, S62610A, S62610B, S62611A, S62611B, S62612A, S62612B, S62613A, S62613B, S62614A, S62614B, S62615A, S62615B, S62616A, S62616B, S62616D, S62617A, S62617B, S62617G, S62618A, S62620B, S62621A, S62621B, S62622A, S62622B, S62623A, S62623B, S62624A, S62624B, S62625A, S62625B, S62626A, S62626B, S62627A, S62627B, S62630A, S62630B, S62631A, S62631B, S62632A, S62632B, S62633A, S62633B, S62633S, S62634A, S62634B, S62635A, S62635B, S62636A, S62637A, S62637B, S62640B, S62642B, S62643B, S62645B, S62646A, S62646D, S62647A, S62652A, S62654A, S62654B, S62660A, S62660B, S62661A, S62664A, S62665A, S6292XA, S63005A, S63014A, S63015A, S63022A, S63024A, S63025A, S63044A, S63054A, S63064A, S63075A, S63092A, S63094A, S63095A, S63112A, S63115A, S63144A, S63235A, S63237A, S63255A, S63260A, S63261A, S63262A, S63264A, S63266A, S63267A, S63273A, S63282A, S63283A, S63284A, S63285A, S63286A, S63294A, S63295A, S63322A, S63391A, S63392A, S63416A, S63501A, S63501D, S63502A, S63591A, S63592A, S63602A, S63610A, S63615A, S63617A, S63630A, S63632A, S63633A, S638X2A, S6391XA, S6392XA, S6401XA, S6402XA, S6411XA, S6412XA, S6421XA, S6422XA, S6431XA, S6432XA, S64490A, S64491A, S64492A, S64493A, S64494A, S64495A, S64496A, S64497A, S648X1A, S648X2A, S6491XS, S65011A, S65012A, S65091A, S65092A, S65111A, S65112A, S65191S, S65192A, S65292A, S65312A, S65505A, S65507A, S65510A, S65511A, S65512A, S65513A, S65514A, S65515A, S65516A, S65517A, S65518A, S65591A, S65593A, S65598A, S65812A, S65901A, S65911A, S66021A, S66022A, S66111A, S66113A, S66117A, S66120A, S66121A, S66122A, S66123A, S66124A, S66125A, S66126A, S66127A, S66128A, S66221A, S66222A, S66292A, S66303A, S66305A, S66315A, S66320A, S66321A, S66322A, S66323A, S66324A, S66325A, S66326A, S66327A, S66391A, S66393A, S66395A, S66421A, S66521A, S66523A, S66525A, S66811A, S66812A, S66821A, S66822A, S66892A, S6701XA, S6702XA, S67190A, S67191A, S67192A, S67193A, S67194A, S67195A, S67195S, S67196A, S67197A, S6721XA, S6722XA, S6741XA, S6742XA, S68012A, S68021A, S68022A, S68110A, S68112A, S68115A, S68116A, S68117A, S68120A, S68120D, S68121A, S68122A, S68123A, S68124A, S68125A, S68126D, S68127A, S68422A, S68521A, S68522A, S68611A, S68613A, S68614A, S68615A, S68620A, S68621A, S68621S, S68622A, S68623A, S68623S, S68624A, S68625A, S68626A, S68627A, S68711A, S6981XA, S6982XA, S6991XA, S6992XA, S6992XD, S7000XA, S7001XA, S7001XD, S7001XS, S7002XA, S7010XA, S7011XA, S7011XD, S7012XA, S7012XD, S7012XS, S70211A, S70212A, S70219A, S70222A, S70311A, S70312A, S70319A, S70321A, S70322A, S70361A, S70372A, S70921A, S71001A, S71002A, S71011A, S71012A, S71032A, S71101A, S71101S, S71102A, S71111A, S71112A, S71121A, S71122A, S71131A, S71132A, S71141A, S71142A, S71152A, S72001A, S72001D, S72001G, S72001K, S72001P, S72001S, S72002A, S72002D, S72002K, S72002S, S72009A, S72009D, S72009S, S72011A, S72011D, S72012A, S72012D, S72012K, S72031A, S72032A, S72034A, S72035A, S72035D, S72041A, S72042A, S72042D, S72044A, S72045A, S72051A, S72051D, S72052A, S72052D, S72061A, S72062A, S72091A, S72091D, S72091S, S72092A, S72101A, S72101D, S72101K, S72102A, S72102D, S72111A, S72111D, S72111G, S72112A, S72112K, S72112S, S72114A, S72115A, S72121A, S72122A, S72122S, S72135A, S72141A, S72141D, S72141G, S72141K, S72142A, S72142D, S72142K, S72142P, S72142S, S72144A, S72145A, S72145D, S7221XA, S7221XD, S7221XK, S7222XA, S7222XD, S7222XK, S7223XD, S7225XA, S72301A, S72301B, S72301D, S72301G, S72301K, S72302A, S72302B, S72302D, S72302K, S72321A, S72322A, S72331A, S72332A, S72334A, S72341A, S72342A, S72342B, S72351A, S72351B, S72352A, S72352B, S72355A, S72361A, S72362A, S72391A, S72392A, S72392K, S72401A, S72401B, S72401C, S72401D, S72401G, S72401K, S72401P, S72401S, S72402A, S72402B, S72402K, S72402S, S72411A, S72411K, S72412A, S72412B, S72421A, S72421B, S72421G, S72422A, S72422B, S72424A, S72424B, S72424D, S72425A, S72431A, S72431B, S72431G, S72432A, S72432B, S72434A, S72441A, S72442A, S72451A, S72451B, S72451K, S72452A, S72452B, S72452C, S72452K, S72452S, S72462A, S72491A, S72491D, S72491S, S72492A, S72492B, S72492D, S72492K, S72492S, S728X1A, S728X1K, S728X1S, S728X2A, S728X2K, S7290XD, S7291XA, S7291XD, S7291XE, S7292XA, S7292XB, S7292XD, S73001A, S73004A, S73005A, S73005S, S73014A, S73015A, S73034A, S73101A, S73102A, S73109, S73191A, S73191S, S73192A, S7401XA, S7411XA, S7412XA, S75001A, S75002A, S75011A, S75022A, S75091A, S75092A, S75111A, S75119A, S75192A, S75811A, S76001A, S76011A, S76012A, S76091A, S76111A, S76111D, S76112A, S76121A, S76122A, S76191A, S76192A, S76192D, S76211A, S76212A, S76311A, S76312A, S76811A, S76812A, S76821A, S76911A, S76912A, S7711XA, S7712XA, S7722XA, S79811A, S79911A, S79912A, S79921A, S8000XA, S8001XA, S8001XD, S8002XA, S8011XA, S8011XD, S8011XS, S8012XA, S8012XD, S8012XS, S80211A, S80211D, S80212A, S80212D, S80221A, S80222A, S80811A, S80812A, S80812S, S80819A, S80821A, S80821D, S80822A, S80861A, S80862A, S80869A, S80871A, S80871D, S80911A, S80921A, S80922A, S81001A, S81001S, S81002A, S81011A, S81012A, S81012D, S81021A, S81032A, S81041A, S81041D, S81042A, S81052A, S81801A, S81801D, S81801S, S81802A, S81802D, S81802S, S81809A, S81809D, S81811A, S81811D, S81812A, S81812D, S81812S, S81819A, S81821A, S81822A, S81831A, S81832A, S81851A, S81852A, S82001A, S82001B, S82001D, S82002A, S82002B, S82002C, S82002D, S82021A, S82031A, S82032A, S82032B, S82035D, S82041A, S82041B, S82042A, S82042B, S82042E, S82042K, S82044A, S82045A, S82091A, S82091B, S82091D, S82092A, S82092K, S82101A, S82101B, S82101C, S82101D, S82101E, S82101N, S82101P, S82102A, S82102B, S82102C, S82102D, S82102K, S82102M, S82111A, S82115A, S82121A, S82122A, S82131A, S82132A, S82132C, S82135A, S82141A, S82141B, S82141C, S82141D, S82141K, S82141N, S82141P, S82141S, S82142A, S82142B, S82142C, S82142D, S82142E, S82142H, S82142K, S82142N, S82142P, S82142S, S82144A, S82145A, S82145B, S82151A, S82152A, S82152B, S82154A, S82191A, S82191B, S82192A, S82192B, S82192D, S82192Q, S82201A, S82201B, S82201C, S82201D, S82201G, S82201J, S82201K, S82201M, S82201N, S82201S, S82202A, S82202B, S82202D, S82202G, S82202K, S82202M, S82202S, S82221A, S82221B, S82221D, S82222A, S82222B, S82225A, S82231A, S82232A, S82232B, S82234A, S82241A, S82241D, S82241S, S82242A, S82242B, S82242C, S82245A, S82251A, S82251B, S82251C, S82251M, S82252A, S82252B, S82252C, S82261A, S82261B, S82262A, S82262B, S82291A, S82291B, S82291D, S82291N, S82292A, S82292B, S82292C, S82292K, S82301A, S82301B, S82301D, S82301K, S82301M, S82301P, S82301S, S82302A, S82302B, S82302C, S82302E, S82302K, S82302M, S82391A, S82391B, S82391C, S82391G, S82391S, S82392A, S82392B, S82392C, S82401A, S82401B, S82401D, S82401K, S82401M, S82402A, S82402B, S82402D, S82402H, S82402K, S82402S, S82409A, S82421A, S82421B, S82422A, S82422B, S82424A, S82425A, S82431A, S82431B, S82432A, S82432B, S82432D, S82434A, S82435A, S82441A, S82441B, S82441D, S82442A, S82442B, S82444A, S82451A, S82451B, S82451C, S82451D, S82451M, S82452A, S82452B, S82452C, S82453A, S82454A, S82455A, S82461A, S82461B, S82462B, S82491A, S82491B, S82491D, S82492A, S82492B, S82492D, S82492Q, S8251XA, S8251XB, S8251XC, S8251XD, S8251XK, S8252XA, S8252XB, S8252XC, S8252XD, S8254XA, S8255XA, S8261XA, S8261XB, S8261XD, S8261XG, S8261XK, S8262XA, S8262XC, S8262XD, S8262XG, S8264XA, S8264XB, S8265XA, S8265XD, S82831A, S82831B, S82831C, S82831D, S82831G, S82831K, S82831M, S82832A, S82832B, S82832C, S82832D, S82832E, S82832M, S82841A, S82841B, S82841C, S82841D, S82841K, S82842A, S82842B, S82842C, S82842D, S82842E, S82842M, S82844A, S82845A, S82851A, S82851B, S82851C, S82851M, S82852A, S82852B, S82852C, S82852D, S82852G, S82852M, S82852P, S82854A, S82861A, S82862A, S82871A, S82871B, S82871D, S82871E, S82871H, S82871K, S82871M, S82872A, S82872B, S82872C, S82872D, S82872H, S82872K, S82872M, S82872Q, S82872S, S82874A, S82875A, S82891A, S82891B, S82891C, S82891D, S82891K, S82891S, S82892A, S82892B, S82892D, S82892E, S82892K, S82892S, S8291XS, S8292XS, S83002A, S83015A, S83104A, S83105A, S83114A, S83194A, S83195A, S83206A, S83207A, S83209A, S83222A, S83231A, S83232A, S83241A, S83241D, S83242A, S83261A, S83281A, S83281D, S83282A, S8332XA, S83411A, S83411D, S83412A, S83421A, S83422A, S83511A, S83511D, S83512A, S83521A, S83522A, S838X1A, S838X2A, S8390XA, S8391XA, S8392XA, S8411XA, S8412XA, S8491XD, S8492XD, S8492XS, S85012A, S85091A, S85092A, S85102A, S85142A, S85171A, S85172A, S85182A, S85202A, S85212A, S85312A, S86011A, S86011D, S86012A, S86021A, S86091A, S86092A, S86122A, S86211A, S86222A, S86311A, S86322A, S86391A, S86801A, S86811A, S86812A, S86821A, S86822A, S86902A, S86911A, S8701XS, S8702XS, S8780XS, S8781XA, S8782XA, S8782XS, S88111S, S88122A, S88912A, S89001A, S89002A, S89101G, S89102A, S89131A, S89191A, S89192A, S89192D, S89201A, S89202A, S89302A, S89391A, S89391G, S8981XA, S8982XA, S8990XS, S8991XA, S8991XD, S8991XS, S8992XA, S8992XD, S9001XA, S9002XA, S90111A, S90112A, S90121A, S90122A, S90211A, S90222A, S9030XA, S9031XA, S9031XS, S9032XA, S90411A, S90412A, S90414A, S90415A, S90421A, S90422A, S90424A, S90425A, S90511A, S90512A, S90521A, S90522A, S90561A, S90562A, S90811A, S90812A, S90821A, S90822A, S90829A, S90852A, S90872A, S90911A, S90921A, S90922A, S90932A, S91001A, S91002A, S91002D, S91002S, S91011A, S91012A, S91021A, S91022A, S91051A, S91101A, S91102A, S91104S, S91105A, S91109A, S91111A, S91111D, S91112A, S91114A, S91115A, S91119A, S91125A, S91132S, S91135A, S91139A, S91141A, S91152A, S91201A, S91202A, S91202D, S91204A, S91205A, S91209A, S91212A, S91215A, S91301A, S91302A, S91311A, S91312A, S91312D, S91321S, S91322A, S91331A, S91339A, S91341A, S91342A, S91351A, S91352A, S92001A, S92001B, S92001D, S92001K, S92001P, S92001S, S92002A, S92002B, S92002D, S92002G, S92011A, S92012A, S92012B, S92014A, S92021A, S92021B, S92022A, S92025A, S92031A, S92041B, S92042A, S92052A, S92061A, S92061B, S92062A, S92062G, S92065A, S92101A, S92101B, S92101P, S92102A, S92102B, S92102D, S92102K, S92111A, S92111B, S92112A, S92112D, S92114A, S92115A, S92115B, S92121A, S92121B, S92121D, S92121S, S92122A, S92131A, S92132A, S92141A, S92142A, S92145A, S92151B, S92152A, S92152B, S92155A, S92191A, S92191B, S92192A, S92192B, S92192D, S92201A, S92211A, S92211B, S92212A, S92215B, S92221B, S92222A, S92231B, S92232A, S92234A, S92235A, S92241A, S92241B, S92242A, S92244A, S92245A, S92251A, S92251B, S92251D, S92252A, S92255A, S92301A, S92302A, S92302D, S92311A, S92311B, S92311G, S92312A, S92314A, S92315A, S92321A, S92321B, S92322A, S92322D, S92324A, S92324B, S92324D, S92325A, S92325B, S92331A, S92331B, S92331G, S92332A, S92332B, S92334A, S92334B, S92334D, S92335A, S92341A, S92341D, S92341G, S92342A, S92342B, S92342D, S92342K, S92344A, S92344B, S92344D, S92345A, S92351A, S92351D, S92351G, S92351K, S92352A, S92352B, S92352D, S92352K, S92353A, S92354A, S92354B, S92355A, S92401A, S92401D, S92402A, S92402B, S92402D, S92405A, S92411A, S92411B, S92412A, S92412B, S92414A, S92416A, S92421A, S92421B, S92421D, S92422A, S92422B, S92425A, S92492A, S92501D, S92502A, S92511A, S92512A, S92512D, S92514A, S92515A, S92522A, S92531A, S92532B, S92534A, S92591A, S92592A, S92811A, S92901D, S92902B, S92902D, S92909A, S92911A, S92911B, S92911D, S92912A, S92912D, S92919D, S9302XA, S9304XA, S9305XA, S93104A, S93115A, S93121A, S93122A, S93125A, S93139A, S93302A, S93312A, S93314A, S93315A, S93325A, S93325D, S93326A, S93332A, S93335A, S93401A, S93401D, S93402A, S93402D, S93402S, S93409A, S93409D, S93421A, S93421D, S93422A, S93431A, S93431D, S93432A, S93491A, S93492A, S93501A, S93601A, S93602A, S93691A, S93699A, S9421XA, S9422XA, S9422XD, S948X1A, S9492XA, S96011A, S96111A, S96122A, S96811A, S96811D, S96812A, S96821A, S96891A, S96911A, S9701XA, S9702XA, S97112A, S97121A, S97122A, S9781XA, S9781XD, S9782XA, S98322A, S99811A, S99812A, S99821A, S99822A, S99911A, S99911S, S99912A, S99921A, S99921S, S99922A, S99929A, T148, T148XXA, T148XXD, T148XXS, T1490, T1490XA, T1490XS, T1491, T1491XA, T2000XA, T2005XS, T2017XA, T2029XA, T2035XA, T2101XA, T2101XS, T2102XA, T2102XD, T2102XS, T2104XA, T2104XD, T2105XA, T2106XD, T2107XA, T2111XA, T2112XA, T2121XA, T2122XA, T2123XA, T2133XA, T2134XA, T2135XA, T22012A, T22041A, T22111A, T22112A, T22131A, T22132A, T22151A, T22211A, T22211D, T22212A, T22221A, T23001D, T23002A, T23011A, T23012A, T23021D, T23021S, T23022S, T23031A, T23032S, T23071A, T23121A, T23131A, T23132A, T23201A, T23202A, T23211A, T23211D, T23221A, T23231A, T23231D, T23232A, T23311D, T23321A, T23322A, T23712A, T24001D, T24001S, T24002A, T24002S, T24011A, T24011D, T24012A, T24022A, T24031S, T24032S, T24102A, T24202A, T24202D, T24211A, T24212A, T24311A, T24312A, T24611A, T25012A, T25012D, T25021A, T25022A, T25022S, T25122A, T25222A, T25222S, T25331A, T2691XA, T270XXA, T280XXA, T282XXA, T285XXA, T286XXA, T286XXS, T287XXA, T300, T310, T3111, T320
