# Supplementary figures and images for: Association between albumin-corrected anion gap and in-hospital mortality of intensive care patients with trauma: A retrospective study based on MIMIC-Ⅲ and Ⅳ databases
Source: PLoS One. 2024 Mar 7;19(3):e0300012. doi: 10.1371/journal.pone.0300012 (PMC10919588; doi:10.1371/journal.pone.0300012)

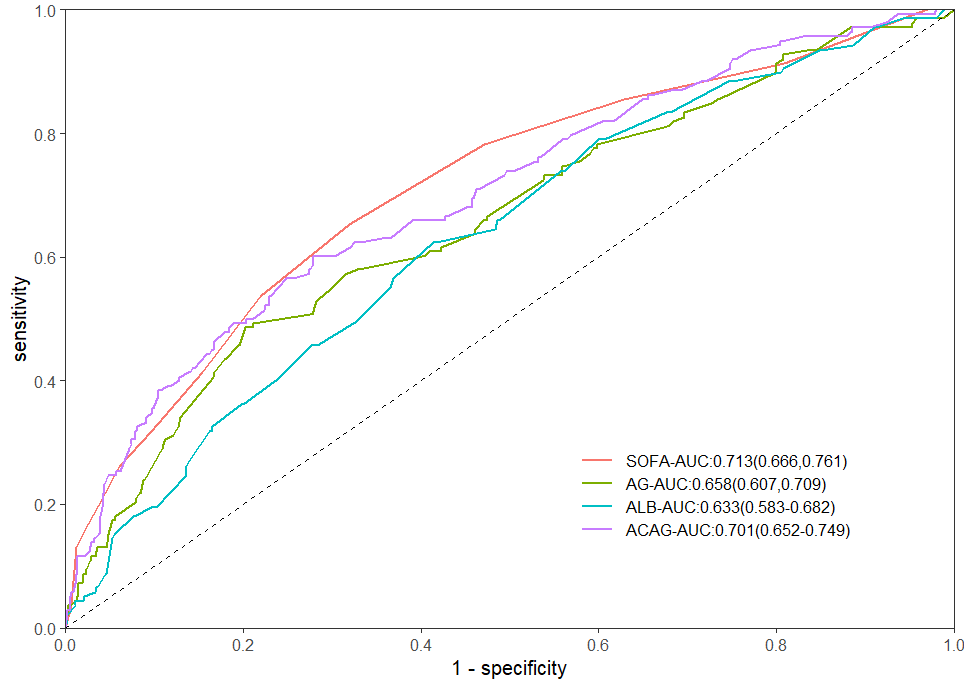

Supplement: S1 Data — (ZIP) [file pone.0300012.s001.zip › Raw data/Figures/ROC.tif]

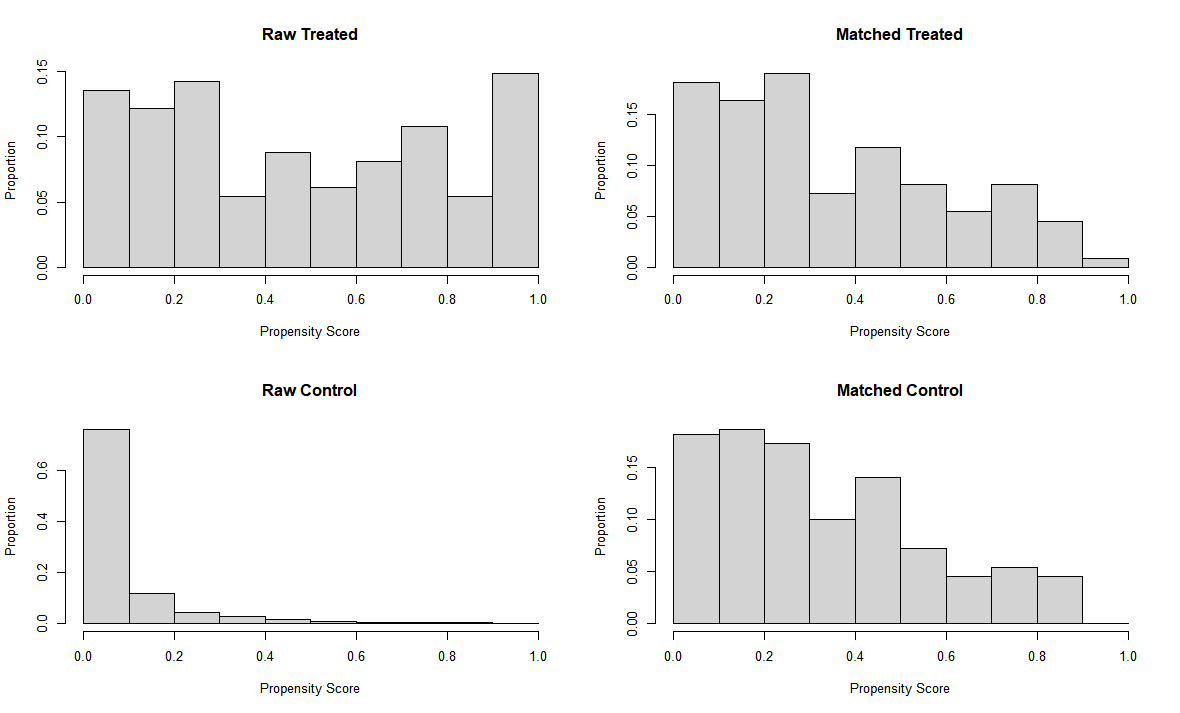

Supplement: S1 Data — (ZIP) [file pone.0300012.s001.zip › Raw data/Figures/hist.tif]

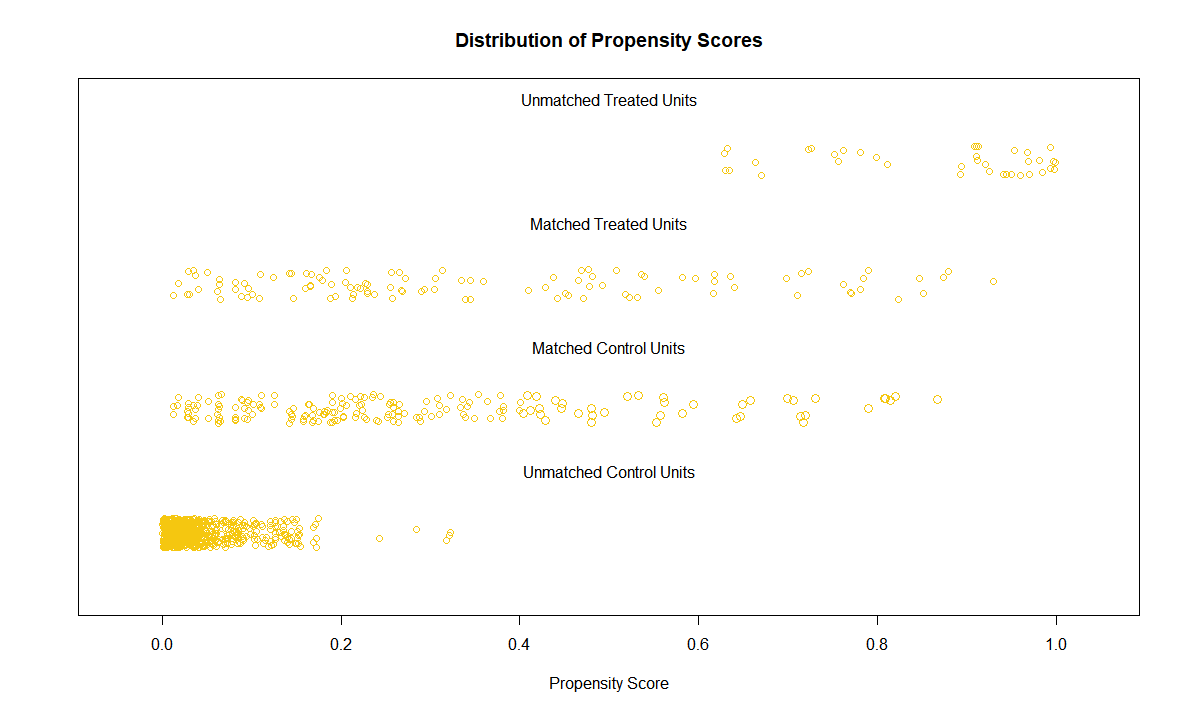

Supplement: S1 Data — (ZIP) [file pone.0300012.s001.zip › Raw data/Figures/jitter.tif]

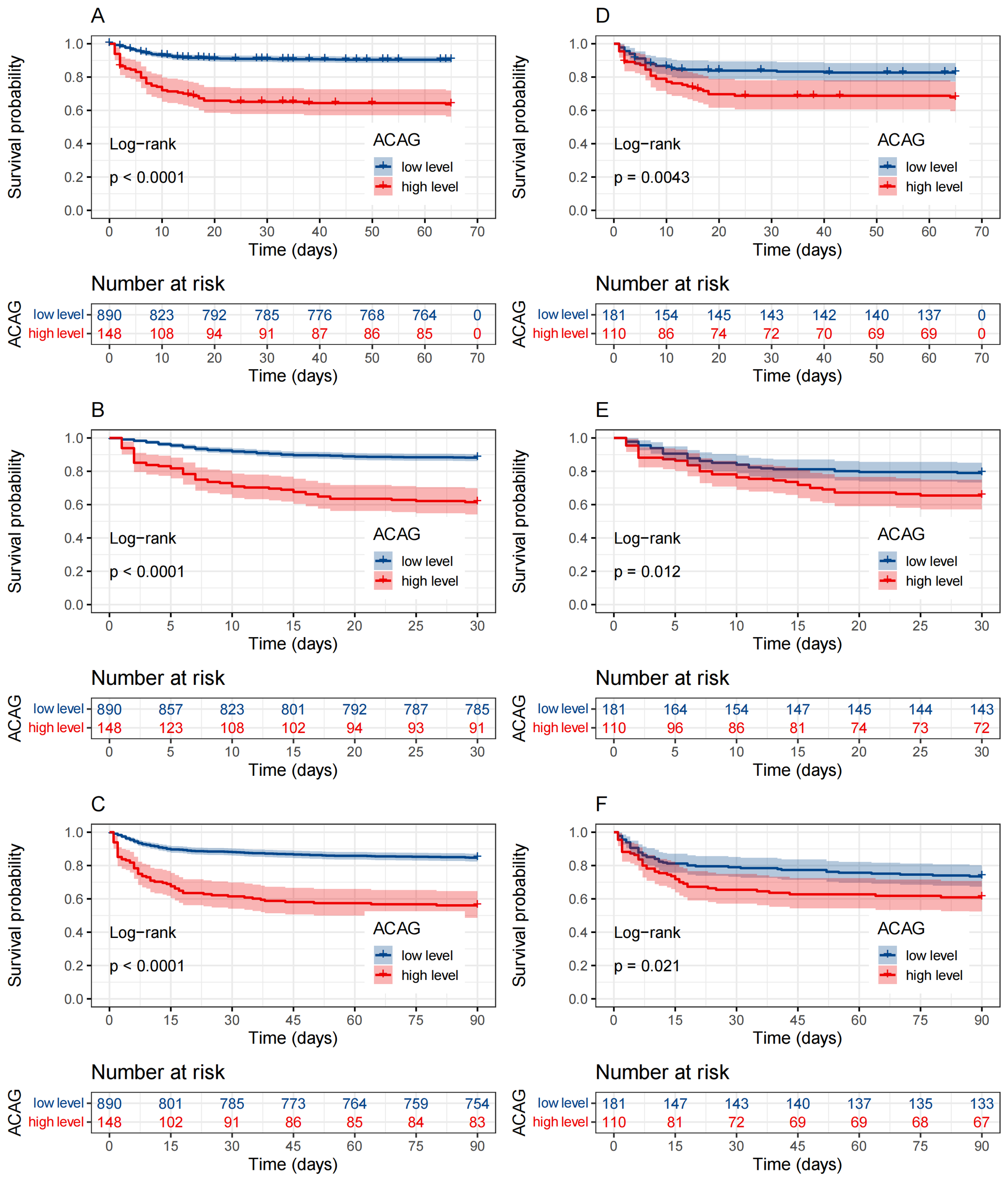

Supplement: S1 Data — (ZIP) [file pone.0300012.s001.zip › Raw data/Figures/km.tif]

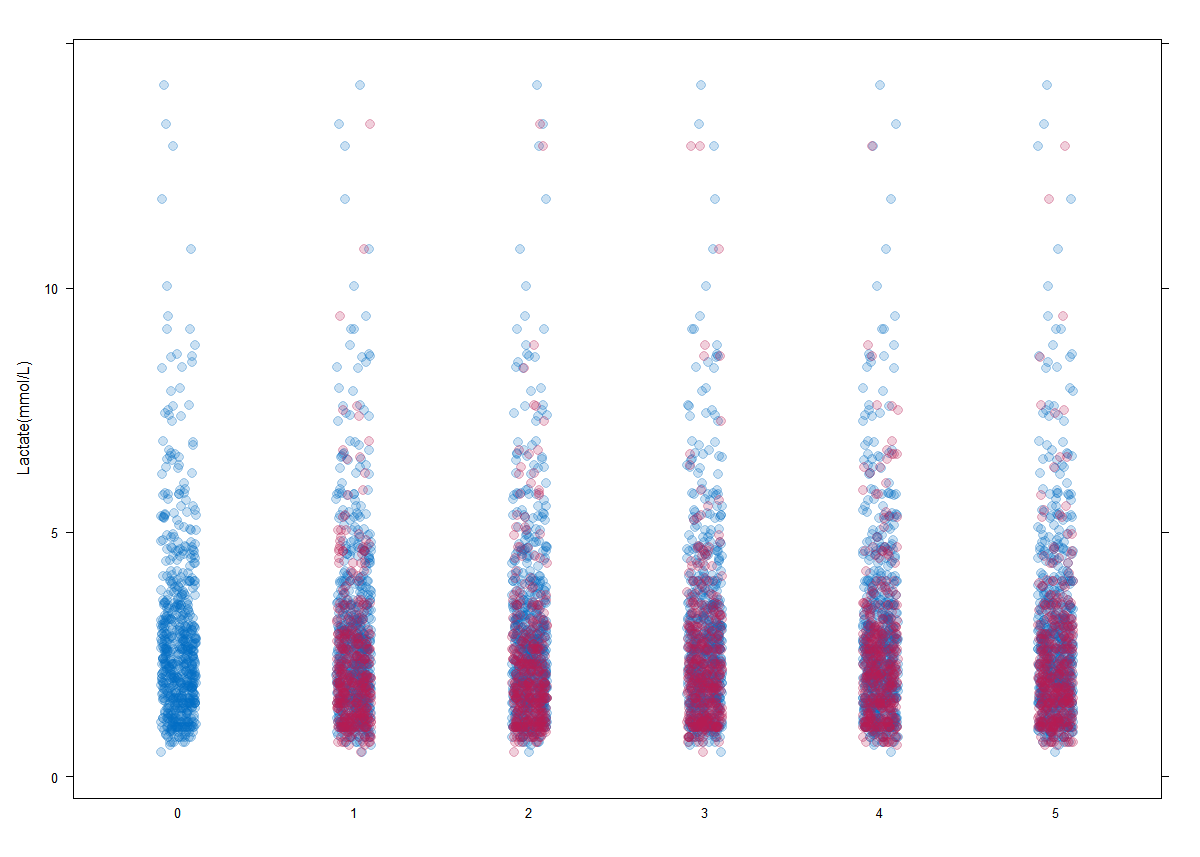

Supplement: S1 Data — (ZIP) [file pone.0300012.s001.zip › Raw data/Figures/lactate_mice.tif]

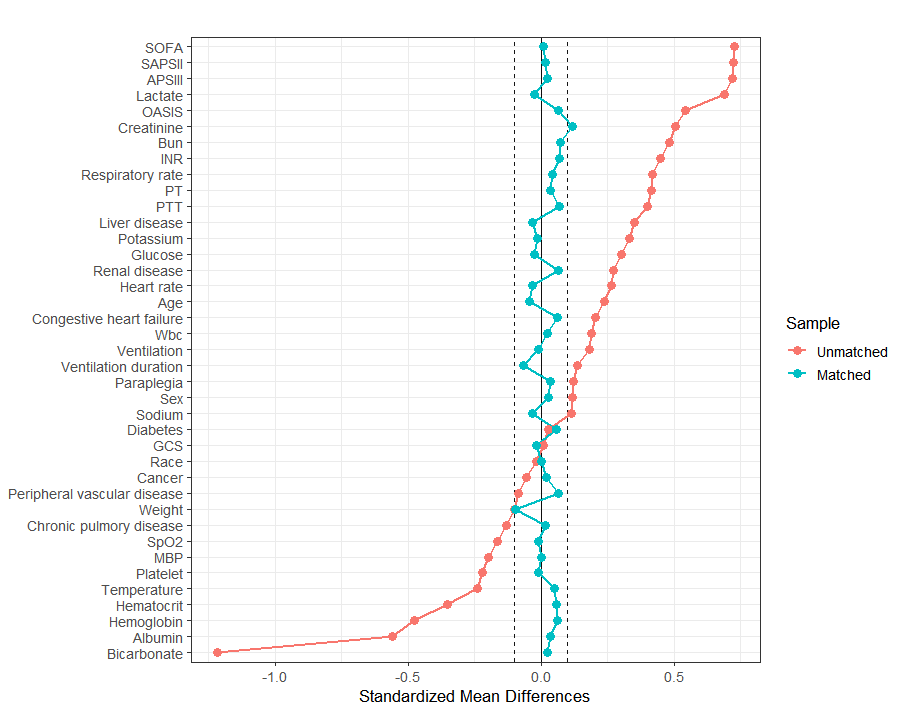

Supplement: S1 Data — (ZIP) [file pone.0300012.s001.zip › Raw data/Figures/smd.tif]
