# Supplementary material for: Association between albumin-corrected anion gap and in-hospital mortality of intensive care patients with trauma: A retrospective study based on MIMIC-Ⅲ and Ⅳ databases
Source: PLoS One. 2024 Mar 7;19(3):e0300012. doi: 10.1371/journal.pone.0300012 (PMC10919588; doi:10.1371/journal.pone.0300012)
Supplement: S1 File — (DOCX) [file pone.0300012.s003.docx]

**Table S1** Cox proportional hazard analysis of ACAG in the data set excluding cases with missing lactate values

| Variable | Crude | | Model 1 | | Model 2 | |
| --- | --- | --- | --- | --- | --- | --- |
|  | HR(95% CI) | P | HR(95% CI) | P | HR(95% CI) | P |
| ACAG<20.357mmol/L | 1(ref) |  | 1(ref) |  | 1(ref) |  |
| ACAG≥20.357mmol/L | 4.609(3.168-6.707) | <0.001 | 4.365(2.937-6.486) | <0.001 | 3.259(1.536-6.915) | 0.002 |
| Continuous | 1.195(1.145-1.248) | <0.001 | 1.161(1.109-1.216) | <0.001 | 1.146(1.049-1.252) | 0.004 |

Crude: No covariates were adjusted. Model1: adjusted for age, race, sex, liver disease, congestive heart failure, renal disease, cancer, diabetes. Model2: adjusted for age, race, sex, MBP, respiratory rate, SpO2, SOFA, SAPSⅡ, APSⅢ, OASIS, hematocrit, hemoglobin, platelets, albumin, anion gap, bicarbonate, bun, creatinine, sodium, lactate, INR, PT, PTT, glucose, liver disease, congestive heart failure, renal disease, cancer, diabetes, ventilation, ventilation duration. ACAG:albumin corrected anion gap.

**Table S2** Training set and Testing set

| **Variables** | **Overall** | **Training Set** | **Testing Set** | ***P*.overall** |
| --- | --- | --- | --- | --- |
| N | 1038 | 726 | 312 |  |
| Age (year) | 58.36 [39.19, 74.59] | 58.20 [40.03, 73.86] | 58.51 [38.00, 75.87] | 0.721 |
| Female, n(%) | 335 (32.3) | 230 (31.7) | 105 (33.7) | 0.582 |
| Weight (kg) | 76.50 [65.00, 89.10] | 77.00 [65.00, 89.25] | 74.95 [63.60, 89.03] | 0.302 |
| Race, n(%) |  |  |  | 0.052 |
| Others | 267 (25.7) | 188 (25.9) | 79 (25.3) |  |
| White | 656 (63.2) | 469 (64.6) | 187 (59.9) |  |
| Black | 39 ( 3.8) | 27 ( 3.7) | 12 ( 3.8) |  |
| Hispanic | 55 ( 5.3) | 32 ( 4.4) | 23 ( 7.4) |  |
| Asian | 21 ( 2.0) | 10 ( 1.4) | 11 ( 3.5) |  |
| *Vital signs* |  |  |  |  |
| Heart rate (bpm) | 86.07 [75.66, 100.00] | 86.92 [76.01, 100.97] | 85.48 [75.05, 97.54] | 0.231 |
| MBP (mmHg) | 81.08 [74.22, 88.31] | 80.99 [74.27, 88.73] | 81.49 [74.20, 87.65] | 0.474 |
| Respiratory rate (bpm) | 18.25 [16.37, 20.70] | 18.34 [16.38, 20.86] | 18.16 [16.36, 20.27] | 0.531 |
| Temperature (℃) | 37.06 [36.73, 37.47] | 37.07 [36.73, 37.47] | 37.05 [36.73, 37.46] | 0.787 |
| SpO2 (%) | 98.14 [96.79, 99.30] | 98.17 [96.79, 99.33] | 98.00 [96.85, 99.23] | 0.614 |
| *Scoring systems* |  |  |  |  |
| GCS | 15.00 [13.00, 15.00] | 15.00 [13.00, 15.00] | 15.00 [13.00, 15.00] | 0.561 |
| SOFA | 4.00 [2.00, 6.00] | 4.00 [2.00, 6.00] | 4.00 [2.00, 6.00] | 0.842 |
| SAPSⅡ | 31.00 [23.00, 41.00] | 31.00 [23.00, 40.00] | 31.00 [23.00, 41.25] | 0.922 |
| APSⅢ | 38.00 [28.00, 52.00] | 39.00 [28.00, 52.00] | 38.00 [28.00, 52.00] | 0.920 |
| OASIS | 32.00 [27.00, 38.00] | 32.00 [27.00, 37.00] | 32.00 [27.00, 38.00] | 0.666 |
| *Laboratory parameters* |  |  |  |  |
| Hematocrit (%) | 33.24 [29.47, 37.37] | 33.30 [29.45, 37.43] | 32.93 [29.54, 37.33] | 0.769 |
| Hemoglobin (g/dl) | 11.50 [10.07, 12.89] | 11.53 [10.05, 12.90] | 11.36 [10.12, 12.80] | 0.491 |
| Platelet (10^9/L) | 192.67 [144.57, 241.50] | 194.00 [148.35, 241.50] | 190.60 [136.38, 241.12] | 0.220 |
| Wbc (10^9/L) | 11.27 [8.40, 14.44] | 11.25 [8.41, 14.39] | 11.40 [8.40, 14.50] | 0.804 |
| Albumin (g/dl) | 3.40 [2.90, 3.80] | 3.40 [2.90, 3.80] | 3.50 [3.00, 3.80] | 0.216 |
| Anion gap (mmol/L) | 14.10 [12.21, 16.00] | 14.00 [12.04, 16.00] | 14.29 [12.33, 16.27] | 0.531 |
| ACAG (mmol/L) | 16.50 [14.75, 18.75] | 16.52 [14.75, 18.75] | 16.50 [14.75, 18.75] | 0.892 |
| Bicarbonate (mmol/L) | 23.00 [20.84, 25.46] | 23.00 [21.00, 25.31] | 23.00 [20.67, 25.50] | 0.837 |
| Bun (mg/dL) | 14.00 [10.00, 20.15] | 14.00 [10.00, 19.67] | 14.50 [10.00, 21.50] | 0.356 |
| Creatinine (mg/dL) | 0.83 [0.70, 1.10] | 0.83 [0.68, 1.05] | 0.85 [0.70, 1.13] | 0.233 |
| Sodium (mmol/L) | 139.50 [137.00, 141.67] | 139.50 [137.00, 141.67] | 139.50 [137.50, 141.67] | 0.486 |
| Potassium (mmol/L) | 4.02 [3.74, 4.37] | 4.00 [3.75, 4.37] | 4.05 [3.73, 4.37] | 0.665 |
| Lactate(mmol/L) | 2.30 [1.55, 3.30] | 2.30 [1.50, 3.30] | 2.30 [1.60, 3.33] | 0.586 |
| INR | 1.20 [1.10, 1.34] | 1.20 [1.10, 1.35] | 1.18 [1.10, 1.30] | 0.302 |
| PT | 13.30 [12.20, 14.70] | 13.33 [12.25, 14.77] | 13.27 [12.20, 14.50] | 0.327 |
| PTT | 27.64 [25.20, 31.30] | 27.86 [25.21, 31.65] | 27.35 [24.95, 30.21] | 0.063 |
| Glucose (mg/dL) | 133.00 [112.89, 159.25] | 134.22 [113.00, 159.94] | 131.50 [112.00, 157.25] | 0.429 |
| *Comorbidities* |  |  |  |  |
| Liver disease (%) | 90 ( 8.7) | 61 ( 8.4) | 29 ( 9.3) | 0.728 |
| Paraplegia (%) | 43 ( 4.1) | 31 ( 4.3) | 12 ( 3.8) | 0.885 |
| Chronic pulmory disease (%) | 132 (12.7) | 97 (13.4) | 35 (11.2) | 0.396 |
| Congestive heart failure (%) | 119 (11.5) | 80 (11.0) | 39 (12.5) | 0.562 |
| Peripheral vascular disease (%) | 32 ( 3.1) | 26 ( 3.6) | 6 ( 1.9) | 0.222 |
| Renal disease (%) | 69 ( 6.6) | 41 ( 5.6) | 28 ( 9.0) | 0.066 |
| Cancer (%) | 382 (36.8) | 254 (35.0) | 128 (41.0) | 0.075 |
| Diabetes (%) | 430 (41.4) | 288 (39.7) | 142 (45.5) | 0.092 |
| *Treatment* |  |  |  |  |
| Ventilation (%) | 649 (62.5) | 458 (63.1) | 191 (61.2) | 0.617 |
| Ventilation duration (hours) | 18.00 [0.00, 92.00] | 18.27 [0.00, 93.00] | 15.31 [0.00, 86.69] | 0.621 |
| *Outcomes* |  |  |  |  |
| Length of hospital stays (days) | 8.73 [4.86, 16.69] | 8.85 [4.92, 16.72] | 8.35 [4.80, 16.63] | 0.286 |
| Length of icu stays (days) | 3.24 [1.78, 7.54] | 3.34 [1.81, 7.66] | 2.94 [1.75, 7.06] | 0.267 |
| in-hospital mortality, n(%) | 138 (13.3) | 95 (13.1) | 43 (13.8) | 0.839 |
| 30-day mortality, n(%) | 162 (15.6) | 115 (15.8) | 47 (15.1) | 0.824 |
| 90-day mortality, n(%) | 201 (19.4) | 141 (19.4) | 60 (19.2) | 1.000 |

The dataset was hierarchically divided into a training set and a testing set in a 7:3 ratio. There were no statistically significant differences observed between the two groups. MAP: mean arterial pressure. Wbc: white blood cell. INR: international normalized ratio. PT: prothrombin Time. PTT: partial thromboplastin time. GCS: Glasgow Coma Scale. SOFA: Sequential Organ Failure Assessment. SAPS Ⅱ: Simplified Acute Physiology Scores Ⅱ. APS Ⅲ: Acute Physiology Score Ⅲ. OASIS: Oxford Acute Severity of Illness Score.

**Table S3** Cox proportional hazard analysis of ACAG in the training set and testing set

| Variable | Crude | | Model 1 | | Model 2 | |
| --- | --- | --- | --- | --- | --- | --- |
|  | HR(95% CI) | P | HR(95% CI) | P | HR(95% CI) | P |
| Training set |  |  |  |  |  |  |
| ACAG<20.357mmol/L | 1(ref) |  | 1(ref) |  | 1(ref) |  |
| ACAG≥20.357mmol/L | 4.049(2.661,6.162) | <0.001 | 4.045(2.579,6.344) | <0.001 | 3.147(1.699-5.827) | <0.001 |
| Continuous | 1.219(1.158,1.283) | <0.001 | 1.193(1.125,1.264) | <0.001 | 1.129(1.017-1.252) | 0.023 |
| Testing set |  |  |  |  |  |  |
| ACAG<20.357mmol/L | 1(ref) |  | 1(ref) |  | 1(ref) |  |
| ACAG≥20.357mmol/L | 5.592(3.061,10.217) | <0.001 | 5.837(2.993,11.381) | <0.001 | 4.708(1.743,12.715) | 0.002 |
| Continuous | 1.205(1.125,1.290) | <0.001 | 1.186(1.101,1.279) | <0.001 | 1.172(1.006,1.366) | 0.042 |

Crude: No covariates were adjusted. Model1: adjusted for age, race, sex, liver disease, congestive heart failure, renal disease, cancer, diabetes. Model2: adjusted for age, race, sex, MBP, respiratory rate, SpO2, SOFA, SAPSⅡ, APSⅢ, OASIS, hematocrit, hemoglobin, platelets, albumin, anion gap, bicarbonate, bun, creatinine, sodium, lactate, INR, PT, PTT, glucose, liver disease, congestive heart failure, renal disease, cancer, diabetes, ventilation, ventilation duration. ACAG:albumin corrected anion gap.


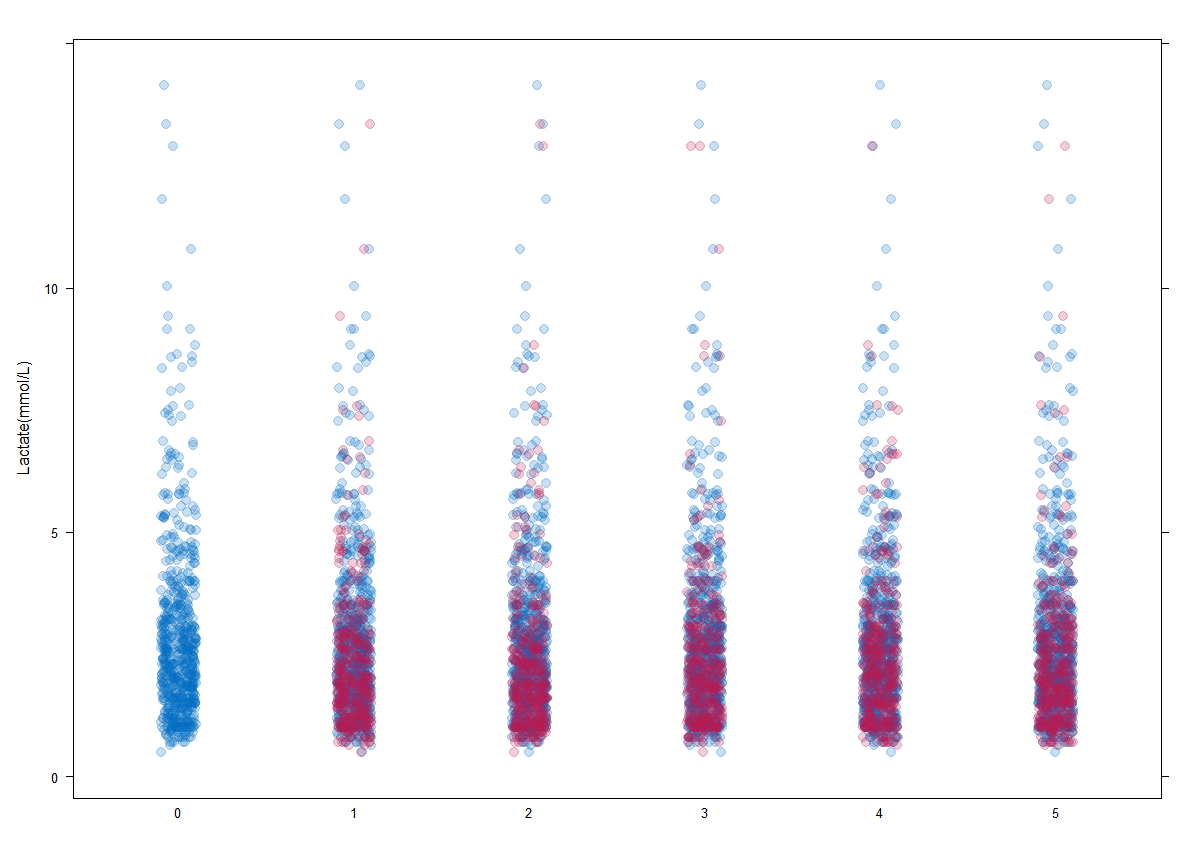


**Figure S1** Multiple imputation scatter plot of lactate. The blue dots represent the observed data, while the red dots indicate the imputed data. The ordinate axis represents the lactate value, and the abscissa axis represents the number of imputations, with 0 representing the original data column.
