# Supplementary material for: Association between albumin-corrected anion gap and in-hospital mortality of intensive care patients with trauma: A retrospective study based on MIMIC-Ⅲ and Ⅳ databases
Source: PLoS One. 2024 Mar 7;19(3):e0300012. doi: 10.1371/journal.pone.0300012 (PMC10919588; doi:10.1371/journal.pone.0300012)
Supplement: Attachment — Submitted filename: Response to ReviewersR2.docx [file pone.0300012.s005.docx]

Dear editor and reviewers of PLOS ONE:

Our reference: PONE-D-23-37440R1

Title: Association between albumin-corrected anion gap and in-hospital mortality of intensive care patients with trauma: A retrospective study based on MIMIC-Ⅲ and Ⅳ databases

By: Yuzhou Xu et al

Thank you very much for your letter and for the editors’ and reviewers’ comments concerning our manuscript entitled "Association between albumin-corrected anion gap and in-hospital mortality of intensive care patients with trauma: A retrospective study based on MIMIC-Ⅲ and Ⅳ databases" (ID: PONE-D-23-37440R1). These comments are of great reference value to the revision and improvement of our paper and have important guiding significance to our researches. We have studied comments carefully and have made correction. We hope that the revision is acceptable and look forward to hearing from you soon. Revised portion are marked in color in the paper. The main corrections in the paper and the responds to the reviewer’s comments are as flowing:

Reviewer 1

1. I am rather glad to review the new version of the manuscript which is much more acceptable for publication. One minor problem remained that the detailed ICD code for trauma or the specific spectrum for the alleged trauma population has been given. The authors are strongly encouraged to provide detailed ICD-9/10 they used to extract the patients on the Github repo or submit to the journal as supplementary materials. Without enough illustration, the conclusions of this article can hardly be put into clinical practice.

We appreciate your valuable feedback, and as per your suggestion, we have incorporated the coding range for trauma-related ICD-9/10 in our article. Specifically, the ICD-9 trauma codes encompass the range of 800-959, while the ICD-10 trauma codes include S00-S99, T00-T14, and T20-T32[1-3].

A comprehensive inventory of trauma-related ICD-9/10 codes within the MIMIC-III/IV databases is provided in the appendix. Furthermore, we have incorporated all diagnostic codes for each patient into the original dataset. The aforementioned items have been submitted to the journal as supplementary materials..

References

[1] Flynn-O'Brien KT, Fallat ME, Rice TB, et al. Pediatric Trauma Assessment and Management Database: Leveraging Existing Data Systems to Predict Mortality and Functional Status after Pediatric Injury. J Am Coll Surg. 2017;224(5):933-944.e5. doi:10.1016/j.jamcollsurg.2017.01.061

[2] Clark DE, Black AW, Skavdahl DH, Hallagan LD. Open-access programs for injury categorization using ICD-9 or ICD-10. Inj Epidemiol. 2018;5(1):11-18. doi:10.1186/s40621-018-0149-8

[3] Wada T, Yasunaga H, Yamana H, et al. Development and validation of a new ICD-10-based trauma mortality prediction scoring system using a Japanese national inpatient database. Inj Prev. 2017;23(4):263-267. doi:10.1136/injuryprev-2016-042106

Furthermore, we have rectified certain formatting issues in the references. All accompanying figures have been adjusted by PACE.

Once again, thank you very much for your comments and suggestions.

Yours sincerely,

Yuzhou Xu
2024-02-04
